# Population Abundance and Ecosystem Service Provision: The Case of Birds

**DOI:** 10.1093/biosci/biy005

**Published:** 2018-03-07

**Authors:** Kevin J Gaston, Daniel T C Cox, Sonia B Canavelli, Daniel García, Baz Hughes, Bea Maas, Daniel Martínez, Darcy Ogada, Richard Inger

**Affiliations:** 1 Environmental and Sustainability Institute at the University of Exeter, in Penryn, Cornwall, United Kingdom; 2 National Institute of Agricultural Technology (INTA) Parana Experimental Station, in Entre Rios, Argentina; 3 Department of Organism and System Biology and the Biodiversity Research Unit at Oviedo University, in Asturias, Spain; 4 Wildfowl and Wetlands Trust at the Slimbridge Wetland Centre, in Gloucestershire, United Kingdom; 5 Department of Botany and Biodiversity Research, Division of Conservation Biology, Vegetation Ecology, and Landscape Ecology, at the University of Vienna, Austria; 6 Africa programs at The Peregrine Fund, in Boise, Idaho, and a research associate at the National Museums of Kenya, in Nairobi

**Keywords:** ecosystem benefits, ecosystem disservices, functional relationships

## Abstract

Although there is a diversity of concerns about recent persistent declines in the abundances of many species, the implications for the associated delivery of ecosystem services to people are surprisingly poorly understood. In principle, there are a broad range of potential functional relationships between the abundance of a species or group of species and the magnitude of ecosystem-service provision. Here, we identify the forms these relationships are most likely to take. Focusing on the case of birds, we review the empirical evidence for these functional relationships, with examples of supporting, regulating, and cultural services. Positive relationships between abundance and ecosystem-service provision are the norm (although seldom linear), we found no evidence for hump-shaped relationships, and negative ones were limited to cultural services that value rarity. Given the magnitude of abundance declines among many previously common species, it is likely that there have been substantial losses of ecosystem services, providing important implications for the identification of potential tipping points in relation to defaunation resilience, biodiversity conservation, and human well-being.

The global biodiversity crisis is not simply one of species loss. Compilations of historical data and the results of monitoring programs are repeatedly revealing major and ongoing local, regional, and global declines in the numbers of individuals of many of those species that do persist (e.g., Inger et al. 2015, Hayhow et al. 2016, WWF 2016, Young et al. 2016). With some notable exceptions (e.g., Markandya et al. 2008, Costello et al. 2016, Naidoo et al. 2016), the consequences of these declines for ecosystem functions, processes, and, particularly, services remain poorly understood. Although huge amounts of time and resources have been directed at determining the consequences of declining species richness for ecosystem function, processes, and services (e.g., Hooper et al. 2005, Cardinale et al. 2012), rather little attention has been paid to the ecosystem-service impacts of the progressive and sometimes rapid losses of numbers of individuals. Nonetheless, the limited evidence suggests that species abundance is critically important for the delivery of ecosystem services (e.g., Davies et al. 2011, Gaston 2011, Winfree et al. 2015), highlighting the need for a better understanding of the functional relationships between the two.

Whether researchers are considering a single species or a group of related species, the functional relationship between their abundance and the level of provision of a given ecosystem service could take a diversity of different possible forms. For simplicity, we initially discuss these forms assuming all else to be equal (paralleling similar discussions about relationships between species richness and ecosystem function or service provision; summarized by Naeem 1998), although of course there could be a wide range of confounding factors (see subsequent separate case-study sections and overall discussion). In theory, although it seems unlikely, there could be services in which the existence of a single individual is sufficient for maximal provision and there is no change in that provision with increasing numbers of individuals (figure 1a). A linear relationship, in which additional individuals add a constant per capita quantity of further provision of service, seems to be ecologically more likely than this (figure 1b). Obvious and likely frequent departures from linearity would be situations in which additional individuals still bring further but progressively smaller provision of service (perhaps as a consequence of intraspecific or interspecific competition), yielding a curvilinear (figure 1c) and perhaps ultimately an asymptotic relationship (figure 1d). Alternatively, facilitation could take place, such that additional individuals bring further and progressively greater per capita provision of service, although it seems inevitable that there will be some limit to such an exponential pattern (figure 1e). In some cases, there might also be an optimum abundance after which point ecosystem-service provision actually decreases with increasing abundance, such as due to interference between individuals in the delivery of service (figure 1f). Indeed, in the extreme, it is not impossible to envisage entirely negative relationships between abundance and provision of an ecosystem service, such as where that service is associated in some way with the degree of rarity of an organism (figure 1g; a priori, this would seem most likely for cultural services, for some of which rarity is highly valued in many contexts; Gaston 1994).

**Figure 1.**
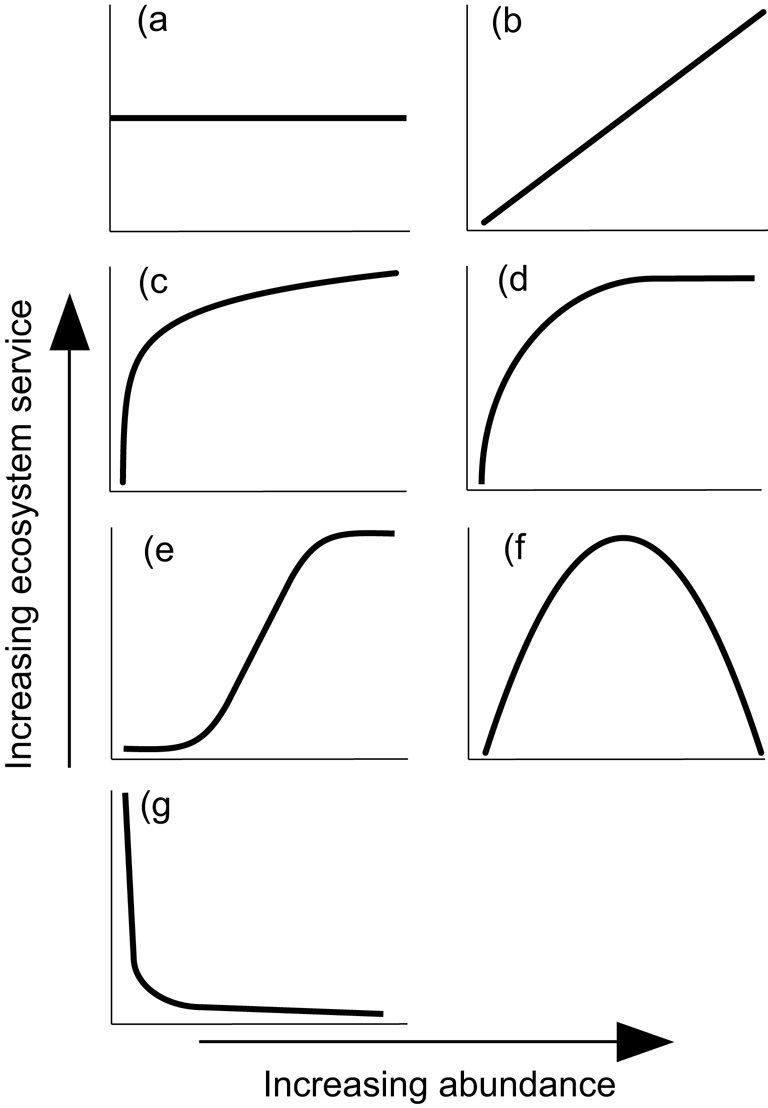
Examples of different potential functional relationships between population abundance and ecosystem-service provision. Relationships are categorized as (a) no relationship, (b) linear, (c) curvilinear, (d) asymptotic, (e) sigmoid, (f) quadratic, and (g) negative curvilinear.

Clearly, the severity of the loss of individuals (of single species or a group of species) will have very different implications for ecosystem-service provision depending on which of these functional relationships prevails in a given circumstance. In this article, we use the case of birds to explore the relationships between organismal abundances and ecosystem services and the actual forms these have been found to take, and we consider the implications. Although doubtless very significant for some, birds are not among the most important providers of many ecosystem services compared with some groups of, say, plants or invertebrates. Nonetheless, they constitute a valuable focal taxon (Șekercioğlu et al. 2016). First, birds do provide a wide range of ecosystem services, including supporting, provisioning, regulating, and cultural services (e.g., Whelan et al. 2008). Second, birds are virtually ubiquitous, occurring in almost all environments, and, commonly being highly mobile, their service provision may extend over wide geographic areas, linking several trophic and physical processes (Šekercioğlu 2006). Finally, they are the most well-studied taxonomic group, with a long and rich history of human interest in birds and the beneficial interactions with people that they provide, yielding important insights even when this has not expressly been formulated in terms of ecosystem services (e.g., Cocker 2014).

## Case studies

We sought studies of functional relationships between bird abundances and ecosystem-service provision, using extensive literature searches (using Web of Science, Google Scholar, and Google; the keywords used included “abundance,” “bird,” and the ecosystem service being searched for, such as “pest control”) and employing stringent criteria when evaluating their suitability (i.e., the study must contain a measure of bird abundance and a measure of ecosystem-service provision). Because many key studies are not identified using such formal processes, our searches were also guided by experts (the authors) in each of the ecosystem services reviewed. Explicit functional relationships have surprisingly seldom been empirically documented, although we found a number of studies with suitable data (a measure of different levels of bird abundance and of associated levels of ecosystem service) that we reanalyzed for this purpose (table 1). Here, for a broad range of different ecosystem services provided by birds, we first propose the theoretical expectation for the associated functional relationships before reviewing the available evidence. The “best” model was determined to be the most parsimonious (based on AIC) with the most variance explained (based on the coefficient of determination).

**Table 1.** The functional shape of relationships between population abundances and ecosystem services—and one disservice—in birds.

Service	Study	Dependent variable	Number of species	Location	Functional shape	n	Parameter 1	Parameter 2	Signif.	R^2^	Suppl. Material
Nutrient transport	Lindeboom 1984	Nitrogen translocation by penguins	2	South Africa	Linear	6	7.67E+06		0.069	0.51	Figure S1a; table S1a
Nutrient transport	Kitchell et al. 1999	Nitrogen translocation by geese	1	United States	Polynomial	15	–1.04E-02	3.91E-07	<0.001***	0.73	Figure 2a; table S1a
Nutrient transport	Fujita and Koike 2009	Nitrogen translocation by crows	1	Japan	Linear	55	2.87E-04		<0.001***	0.85	Figure 2a; table S1a
Seed dispersal	García et al. 2010	Seed dispersal rate by frugivorous birds	6	Spain	Logarithmic	83	1.46E-01		<0.001***	0.46	Figure 2a; table S1b
Seed dispersal	García and Martínez 2012	Seed dispersal rate by frugivorous birds	6	Spain	Logarithmic	89	1.32E-01		<0.001***	0.5	Figure S1a; table S1b
Seed dispersal	Martínez and García 2017	Seed dispersal rate by frugivorous birds	6	Spain	Logarithmic	87	9.21E-02		<0.001***	0.25	Figure S1a; table S1b
Scavenging	This study	Time for vultures to consume carcass	4	Kenya	Asymptotic	49	1.64E+02	–1.66E+01	<0.001***	NA	Figure 2b; table S1c
Scavenging	Inger et al. 2016	Time for crows to consume carcass	1	UK	Asymptotic	62	2.48E+02	3.2E+00	<0.001***	NA	Figure 2b; table S1c
Pest control	Maas et al. 2015	Bite marks on artificial prey	1	Indonesia	Logarithmic	10	3.08 E+01		<0.01**	0.58	Figure 2b; table S1d
Pest control	Crawford and Jennings 1989	Pests consumed by insectivorous birds	25	United States	Linear	22	1.65E+04		<0.001***	0.67	Figure S1b; table S1d
Cultural	This study	Visitor numbers at one WWT site (Welney)	>25	UK	Logarithmic	50	2.14E-01		0.001**	0.20	Figure 2c; table S1e
Cultural	Cox et al. 2017	Bird abundances and depression	>25	UK	Linear	225	–1.06E-02		0.0177*	0.02	Figure S1c; table S1f
Cultural	Cox et al. 2017	Bird abundances and anxiety	>25	UK	Linear	225	–9.92E-03		0.007**	0.02	Figure 2c; table S1f
Cultural	Cox et al. 2017	Bird abundances and stress	>25	UK	Asymptotic	225	3.22E+00	–4.90E+01	<0.001***	NA	Figure 2c; table S1f
Crop damage	Canavelli et al. 2014	Abundance and crop damage	1	Argentina	Polynomial	49	0.52E-01	–1.32E-02	0.0002***	0.25	Figure 3; table S1g

*Note:* See supplemental appendix S1 for statistical analysis and supplemental table S1 for the statistical results and model-selection data.

In general, the empirical studies and reanalyses highlighted here have determined functional relationships between abundance and ecosystem-service provision in the context of multispecies assemblages, so these relationships incorporate competitive and facilitatory intra- and interspecific effects (which have been documented in the context of ecosystem-service provision). In the main, other major influences on the relationships recognized by the investigators were controlled for in the original papers in their respective study designs, and explicit data are not available for us to do so (but see the text for discussion). For consistency and comparability, we present all relationships in their “raw form,” and where information is available, we comment on the consequences of controlling for other variables.

## Nutrient cycling (supporting service)

Animals play important roles in the movement of nutrients (e.g., nitrogen and phosphorus) both within and between ecosystems and habitats. Birds have the potential to contribute substantially to such nutrient flows (Whelan et al. 2008, Bauer and Hoye 2014) and, on average, move nutrients farther than many other taxa. Arguably, attention has focused foremost on the movements of nutrients between marine and terrestrial systems by colonial seabirds (e.g., Polis and Hurd 1996, González-Bergonzoni et al. 2017), with nutrient fluxes being directly associated with increased plant growth (e.g., Anderson and Polis 1999). However, the major seasonal migrations of birds across much of the world also result in shifts in nutrients, including those bound in their own bodies and released in death (Sturges et al. 1974), through their feces (Whelan et al. 2008), and through, for example, increased vegetation decomposition from trampling (Bird et al. 2000). At smaller scales, the movement of nutrients by birds between urban systems and other ecosystems in the wider landscape has been highlighted, particularly because these nutrients may themselves have arrived in towns and cities from afar (given the international nature of much trade in human foodstuffs and that birds will commonly be feeding on the resultant waste; Fujita and Koike 2009). The flow of nutrients can have direct effects, providing a source of fertilizer (Gasket and Smith 2007) and favoring some primary producers over others, and indirect effects, as bottom-up forces cascade to primary consumers (Wootton 1991) and detritivores (Sánchez-Piñero and Polis 2000).

**Figure 2.**
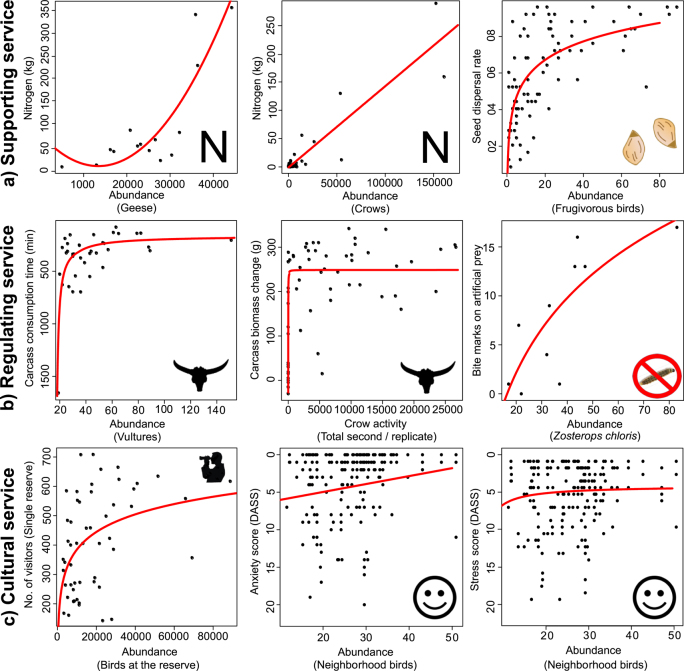
Case studies of relationships between bird abundance and ecosystem-service provision. From left to right, the top row shows supporting services (nutrient transport for geese and crows; seed dispersal); the middle row shows regulating services (scavenging vultures and crows; pest control); and the bottom row shows cultural services (Welney reserve, lower levels of anxiety and stress). See table 1 for details and supplemental figure S1 for plots of further case studies in table 1.

It seems most likely that functional relationships between bird abundance and nutrient deposition will be linear, with a given increase in the number of birds delivering a proportional increase in the movement of nutrients. We found examples for penguins, geese, and crows, and this functional form does generally seem to be the case, although for the geese, the best-fit line was a polynomial (figure 2a; table 1; supplemental table S1). In the penguin study, levels of nitrogen in the rookery (king penguin, *Aptenodytes patagonicus*, and macaroni penguin, *Eudyptes chrysolophus*) increased with the number of individual birds, with degradation of uric acid into ammonia and its subsequent volatilization and deposition increasing nearby plant growth (Lindeboom 1984). In the study of geese (“white geese,” of which 95% were lesser snow geese, *Chen caerulescens caerulescens*, and 5% Ross’ goose, *Chen rossii*), nutrient levels and numbers of both species were measured weekly (Kitchell et al. 1999), with the best-fitting model showing a polynomic increase in nutrient inputs with increasing geese numbers. In the crow study (roosts of *Corvus corone* and *Corvus macrorhynchos*), the nutrient input (as was measured using fecal trays under the canopy) increased linearly with bird abundance (Fujita and Koike 2009).

Of course, a linear increase in nutrient deposition with increasing abundance of birds may well not translate into a linear increase in the effect of this deposition, such as plant productivity, with that growth in abundance. There are likely to be diminishing returns, as well as potentially toxic effects if bird numbers and inputs were sufficient.

## Seed dispersal (supporting service)

Seed dispersal by frugivorous animals is a pivotal ecosystem function and service in many temperate and tropical ecosystems, with the movement of seeds from source plants driving plant gene flow and population dynamics in undisturbed habitats, as well as vegetation recovery in degraded lands (Farwig and Berens 2012). Birds are often key agents in such dispersal, with the potential to move seeds over considerable distances. Studies in tropical and temperate human-dominated landscapes have shown that the number of individuals rather than just the number of frugivorous species was key in driving seed dispersal (García and Martinez 2012, Pejchar et al. 2012). Seed dispersal by birds has been found to be a cost-effective alternative to planting seedlings for forest regeneration in urban areas (Overdyck et al. 2013). Indeed, in Stockholm National Urban Park, Sweden, the costs of replacing oak tree seed dispersal by the Eurasian jay (*Garrulus glandarius*) by planting are up to US$9400 per hectare (Houger et al. 2006).

One might predict a linear increase in seed dispersal rate—or that the dispersal rate might increase at a declining rate—with the abundance of birds. The latter would seem more likely when the availability of seeds for dispersal is limited or at least when the likelihood of fruits or seeds being found and eaten progressively declines. Also, a curvilinear response of seed dispersal is expected when having seeds deposited everywhere in the landscape requires a disproportionately high abundance of avian seed dispersers, because of the typically contagious patterns of seed deposition by birds (e.g., Carlo et al. 2013). Empirical evidence from three studies, each concerning small numbers of frugivorous bird species, exhibits the same curvilinear functional relationship (figure 2a; table 1; supplemental figure S1a). These studies recorded the number of frugivorous birds observed through fall and winter over large montane areas, as well as the occurrence of tree seeds dropped by birds across hundreds of sampling points. Interestingly, the relationships between bird abundance and seed dispersal emerged strongly even after controlling for the large-scale variability in bird richness (one to six species) and habitat cover (García et al. 2010, García and Martínez 2012, Martínez and García 2017).

## Scavenging (regulating service)

Scavenging is key to energy transfer within ecosystems and is potentially responsible for the transfer of more energy between trophic levels than is predation (Wilson and Wolkovich 2011). It also provides a vital service by removing carcasses from the environment, sometimes with implications for human health and that of livestock and pets. Carcass removal appears primarily to be performed by obligate and facultative vertebrate scavengers (DeVault et al. 2011). Of these, avian scavengers (which include corvids, raptors, and gulls) are key in terms of service delivery. For example, within the archipelago of Socotra, off the Horn of Africa, vultures have been estimated to consume 17%–22% of all putrescible waste (Gangoso et al. 2013).

It seems most likely that scavenging is an asymptotic function of avian abundance, with the likelihood of carcasses being detected and then being consumed both initially increasing with the abundance of potential scavengers, but with the benefit of there being more scavengers progressively declining. For empirical data, for Carrion crows (*Corvus corone*) in an urban area of the United Kingdom, the initial rise with increasing abundance is extremely fast, whereas, for example, for vultures (of four species), the initial rise is slower (table 1; figure 2b). In these studies, natural or standardized carcasses were observed in matched environments and the abundance or activity of birds measured along with the consumption of the carcass; carcass removal by birds was largely too fast for other scavengers or decomposers to have any significant impact.

## Pest control (regulating service)

Foraging by birds has the potential to provide a critical service in controlling the numbers of agricultural pests, such as insects and rodents. Indeed, recent studies have shown how excluding birds from agricultural systems leads to greater pest levels, an increase in pest damage, or decreases in yield (or a combination of these impacts). These pest-control effects of birds have been demonstrated in a wide variety of agricultural systems and in different climates, from tropical agroforestry systems to a number of types of forestry (e.g., Marquis and Whelan 1994, Tremblay et al. 2001, Mols and Visser 2007, Van Bael et al. 2008, Maas et al. 2016).

Against this background, it seems inevitable that relationships between bird abundance and impacts on pest numbers or damage will tend to be broadly positive—reflecting the generality of positive functional responses between the density of a prey population and the intake rate of a consumer (Denny 2014). In many cases, it seems likely that the relationships will simply be linear because the abundance of birds never gets sufficiently high relative to that of the pests (usually insects) that either there is interference between the foraging of different individual birds or the pest resource becomes a limiting factor (e.g., figure S1b; tables 1 and S1). Nevertheless, curvilinear or asymptotic functional relationships are also possible after the occurrence of interference, saturation, and prey switching when given resources become insufficiently profitable. One empirical example indeed shows such a pattern (figure 2b; table 1). In this study on avian impact on pest predation in tropical cacao agroforestry, the researchers found that predation rates on insects were not related to overall species diversity or shade management but to the activity of insectivorous birds, particularly a single abundant species; distance to primary forest was also a driver of predation rates (Maas et al. 2015). This study was conducted on ten pesticide-free cacao farms that covered a shade intensity and forest proximity gradient and therefore provides hitherto-rare results on the impact of both local and landscape management factors on avian pest-control services. Because this experimental study was embedded in a 2-year exclosure experiment (Maas et al. 2013), the authors could also control for large-scale spatiotemporal patterns affecting species community composition of birds and arthropods and agricultural productivity, revealing important insights for the methodological design of such studies (i.e., mist netting versus points counts and the importance of forest proximity for avian pest control in cacao and other agroforestry systems).

## Recreational experiences (cultural service)

Birds provide a wide range of cultural services, including as the focus for recreational activities. Indeed, birdwatching, the act of observing, identifying, and/or recording sightings of different species, is perhaps the most readily quantifiable cultural service provided by birds. Globally, it represents the primary form of ecotourism, and in the United States alone, an estimated US $40.9 billion was spent in 2011 on birding equipment and bird-trip-related expenditure (Carver 2013). Watching birds can be an important component of maintaining people's connection to nature in a rapidly urbanizing world (Cox and Gaston 2016) and if managed correctly can have positive conservation outcomes (Șekercioğlu 2002).

Birdwatchers vary widely in their level of interest, from casual visitors to local habitat patches to individuals highly motivated by the opportunity to observe rare or vagrant species. The predicted functional relationships between the number of birdwatchers benefiting and the abundance of birds is likely to depend on the motivation of the former. For the more casual birdwatchers, one might predict either that there would be no directional relationship or that the benefits would increase with the abundance of birds at a site and therefore the general magnitude of the avian spectacle. We examined the relationship between visitor numbers and the abundances of wintering birds at nature reserves in and around Wildfowl and Wetland Trust (WWT) Wetland Centres in the United Kingdom. Considering all nine centers, we found no significant relationship, even when controlling for weather conditions; however, when we considered the centers individually, we found that at one (WWT Welney Wetland Centre), which has few attractions other than wild birds, there was a significant curvilinear relationship (figures 2c and S1c; table 1).

Of course, for many birdwatchers, geographical rarity is more important than abundance, and some may spend significant time, effort, and money to gain sightings of rare individuals of usually vagrant species. Indeed, it seems likely that in many places, these vagrant individuals achieve much more focal attention than would other individuals of the same species where it is resident; however, we are not aware of any empirical studies that actually demonstrate this (although a negative interspecific relationship has been documented between the numbers of birdwatchers observing a bird and the rarity of the species; Booth et al. 2011). Likewise, we are not aware of empirical studies that explore whether the likelihood of birdwatchers finding rare breeding or wintering bird species is dependent on the overall abundance of birds at a site, although this certainly seems likely often to be so given what is known of relationships between species richness and assemblage abundances (Evans et al. 2005).

## Health and well-being (cultural service)

Interactions with nature have been shown to provide a wide array of health and well-being benefits to people, including physical, mental, and social gains (Keniger et al. 2013, Hartig et al. 2014). Which components of nature are most important for these benefits is not well understood. However, interactions with birds have repeatedly been highlighted as contributing (Kaplan 2001, Keniger et al. 2013) because these can be visually and vocally conspicuous, and some can be attracted to feeders to provide positive nature experiences at close proximity (Cox and Gaston 2016). Studies have documented positive relationships between human well-being and real or perceived avian species richness (Fuller et al. 2007, Dallimer et al. 2012, Shwartz et al. 2014).

The need to understand the functional relationship between human health and well-being and the abundance of components of nature has recently been highlighted in the context of establishing the form of nature dose–response relationships (Shanahan et al. 2015). Such relationships are generally thought likely to be positive and asymptotic but may in some cases also feature declining benefits when nature doses are very high (e.g., high densities of some birds causing damage to property, mess, and noise pollution; Rock 2005). The one empirical example that we could locate (Cox et al. 2017), on urban birds, found that their abundances during the afternoon, when individuals are most likely to be experienced by people, were weakly positively correlated to lower levels of human depression, anxiety, and stress (figures 2c and S1c; table 1). These relationships strengthened when vegetation cover and a variety of sociodemographic factors that are predicted also to contribute to variation in human well-being were controlled for (Cox et al. 2017). How widely these results might generalize is unclear.

## Conclusions

Common ecological knowledge suggests that positive functional relationships between bird abundances and ecosystem-service provision are likely to be the norm. The available empirical evidence supports this and also the likelihood that such positive relationships will seldom be linear. These conclusions seem likely to hold for a wide variety of species and taxonomic groups (e.g., Winfree et al. 2015).

**Figure 3.**
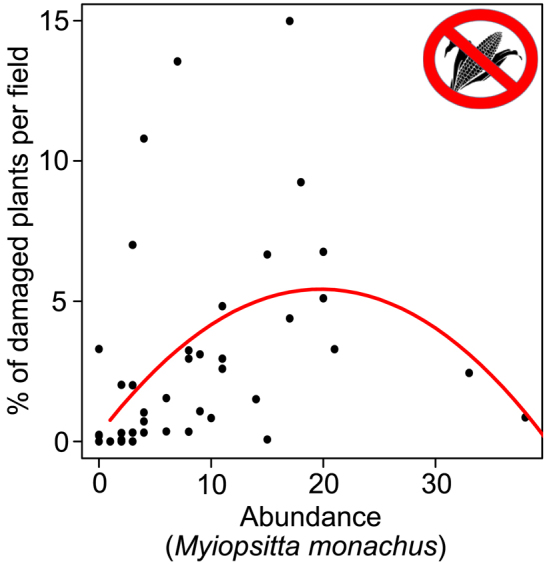
A case study of the relationship between bird abundance and disservice provision (table 1 and supplemental table S1g).

We are not aware of any available empirical evidence for hump-shaped relationships between abundance and ecosystem-service provision, either for birds or for any other group. For birds, this may follow from the relatively narrow spatial and temporal variation that individual species exhibit in local abundances (Gaston 2003), limiting the likelihood that abundances in most cases approach levels at which a service starts to decrease through competition or other intra- or interspecific interactions. However, there is no doubt that, particularly at high numbers, some bird species and combinations of species can also bring about profound ecosystem disservices (e.g., crop destruction) and that these may interact with service provision in complex ways (e.g., geese may fertilize fields while also destroying crops and altering crop yield, which may or may not be the ultimate ecosystem service depending on other benefits of fertilization). The functional relationships between avian abundance and levels of ecosystem disservices are even more poorly documented than are those between abundance and ecosystem services. However, these relationships are probably more likely to be nonlinear than linear, particularly when factors such as social interference or facilitation with other species, aggregation, or the diversity of food availability are taken into account (e.g., see figure 3; Hone 1994).

Negative functional relationships between the abundance of birds and ecosystem-service provision, and likely for other groups, are probably generally limited to some cultural services in which a high value is placed on rarity. This is, however, not a trivial issue, having made for substantial challenges in the conservation of some bird species, for example (Juniper 2002).

Whatever their form, the empirically documented functional relationships illustrate that variation in the abundance of birds can explain substantial variation in ecosystem-service provision (table 1). This is despite the fact that in most of these cases, the effects of additional variables have not been statistically controlled for (including variation in the abundances of other species, of birds, or of other groups). Some of the weakest relationships (e.g., for cultural services) occur where there is little doubt that other such variables are very important. For example, although nature experiences are known to influence human health and well-being, many socioeconomic and other environmental factors at smaller and larger spatial scales influence these, and there is no expectation of strong relationships with nature experiences. Indeed, functional relationships between bird abundances and health outcomes have been shown to strengthen when such other variables are accounted for (Cox et al. 2017).

Three ways of analyzing relationships between biodiversity and ecosystem services have been distinguished: spatial correlations, management comparisons, and functional experiments (Ricketts et al. 2016). This equally applies to relationships between species abundances and ecosystem services, although we note that a correlational approach need not be based only on spatial variation but could equally be based on temporal variation. However, in the case of birds, it is problematic to establish the form of such relationships through management comparisons (because of the challenge of attaining sufficient variation in abundances; Maas et al. 2016) and functional experiments (because of the challenge of conducting such experiments at landscape scales). In consequence, the development of understanding of relationships between abundance and ecosystem services for birds will require either very careful choice of study areas (and/or times)—to minimize the likelihood of marked variation in potential confounding factors—or detailed exploration of the effects of statistical model structures and approaches that account for such factors.

Given the apparent predominance of positive functional relationships between abundances and ecosystem-service provision, it seems likely that as avian populations have recently declined, substantial losses of ecosystem services have occurred. This has principally been as a consequence of losses in the abundances of common species, which are often the major net providers of ecosystem services (even when the provision may be lower per capita than for some rarer species; Gaston 2011). Less-abundant species have in some cases increased in population size (Inger et al. 2015) as a consequence of targeted conservation action (Swinnerton 2001). The shapes of the functional relationships between avian abundances and ecosystem-service provision mean that in some cases, population declines will have quickly led to major losses in that provision (e.g., where functional relationships are linear), whereas in others, there are likely to be thresholds beyond which further declines will lead to catastrophic losses of provision (e.g., strongly asymptotic functional relationships). As such, an improved understanding of the shape of these relationships would contribute to filling critical knowledge gaps on “Anthropocene defaunation” impacts that strongly limit current conservation and management measures (e.g., Dirzo et al. 2014, Socolar et al. 2016), with cascading consequences for various stakeholders. Identifying these causal relationships and baseline parameters will help in formulating and implementing adequate countermeasures as part of an adaptive and scalable management strategy.

The paucity of published studies of the form of relationships between the provision of ecosystem services and the abundances of birds, or indeed any other taxon (Winfree et al. 2015), is surprising. Such relationships are arguably the fundamental building blocks on which those between the provision of ecosystem services and species richness are constructed but which by comparison have attracted a good deal of attention. Closing this gap and determining the relative roles of abundance and species richness in delivering ecosystem services would seem an important research objective at the present time.

## Supplementary Material

Supplemental data
