# Supplementary figures and images for: Population Abundance and Ecosystem Service Provision: The Case of Birds
Source: Bioscience. 2018 Mar 7;68(4):264–72. doi: 10.1093/biosci/biy005 (PMC5905662; doi:10.1093/biosci/biy005)

### a) Supporting service

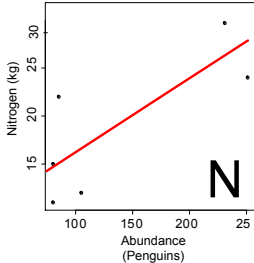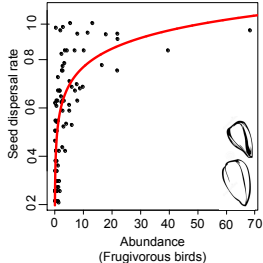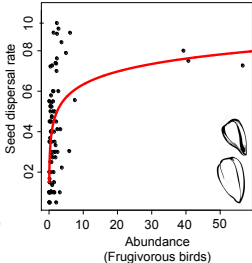

### b) Regulating service

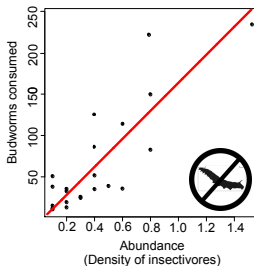

### c) Cultural service

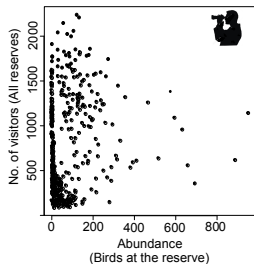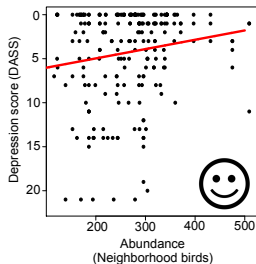

Supplement: Supplemental data [file biy005_supp.pdf]
